# Comparison of two ferritin assay platforms to assess their level of agreement in measuring serum and plasma ferritin levels in patients with chronic kidney disease

**DOI:** 10.1186/s12882-023-03255-6

**Published:** 2023-06-30

**Authors:** Sandawana William Majoni, Jane Nelson, Jessica Graham, Asanga Abeyaratne, David Kiran Fernandes, Sajiv Cherian, Geetha Rathnayake, Jenna Ashford, Lynn Hocking, Heather Cain, Robert McFarlane, Paul Damian Lawton, Federica Barzi, Sean Taylor, Alan Cass

**Affiliations:** 1 Menzies School of Health Research, Charles Darwin University, Northern Territory, Darwin, Australia; 2 Department of Nephrology, Royal Darwin Hospital, Division of Medicine, P.O. Box 41326, Casuarina, Darwin, Northern Territory Australia; 3 Northern Territory Medical Program, Flinders University, Darwin, Northern Territory Australia; 4 Department of Nephrology, Alice Springs Hospital, Alice Springs, Northern Territory Australia; 5 Territory Pathology, Darwin Northern Territory, Darwin, Australia; 6 Territory Pathology, Alice Springs, Northern Territory Australia; 7 Poche Centre for Indigenous Health, The University of Queensland, Brisbane, QLD Australia

## Abstract

**Background:**

Ferritin levels are used to make decisions on therapy of iron deficiency in patients with chronic kidney disease (CKD). Hyperferritinaemia, common among patients with CKD from the Northern Territory (NT) of Australia, makes use of ferritin levels as per clinical guidelines challenging.

No gold standard assay exists for measuring ferritin levels. Significant variability between results from different assays creates challenges for clinical decision-making regarding iron therapy. In the NT, different laboratories use different methods. In 2018, Territory Pathology changed the assay from Abbott ARCHITECT i1000 (AA) to Ortho-Clinical Diagnostics Vitros 7600 (OCD). This was during the planning of the INtravenous iron polymaltose for First Nations Australian patients with high FERRitin levels on haemodialysis (INFERR) clinical trial. The trial design was based on AA assay ferritin levels. We compared the two assays’ level of agreement in measuring ferritin levels in CKD patients.

**Methods:**

Samples from INFERR clinical trial participants were analysed. Other samples from patients whose testing were completed the same day on OCD analyzers and run within 24 h on AA analyzers were added to ensure wide range of ferritin levels, adding statistical strength to the comparison. Ferritin levels from both assays were compared using Pearson’s correlation, Bland–Altman, Deming and Passing-Bablok regression analyses. Differences between sample types, plasma and serum were assessed.

**Results:**

Sixty-eight and 111 (179) samples from different patients from Central Australia and Top End of Australia, respectively, were analyzed separately and in combination. The ferritin levels ranged from 3.1 µg/L to 3354 µg/L and 3 µg/L to 2170 µg/L for AA and OCD assays respectively. Using Bland–Altman, Deming and Passing-Bablok regression methods for comparison, ferritin results were consistently 36% to 44% higher with AA than OCD assays. The bias was up to 49%. AA ferritin results were the same in serum and plasma. However, OCD ferritin results were 5% higher in serum than plasma.

**Conclusions:**

When making clinical decisions, using ferritin results from the same assay in patients with CKD is critical. If the assay is changed, it is essential to assess agreement between results from the new and old assays. Further studies to harmonize ferritin assays are required.

**Supplementary Information:**

The online version contains supplementary material available at 10.1186/s12882-023-03255-6.

## Background

Ferritin levels are used to determine body iron status in making clinical decisions on therapy of iron deficiency in patients with chronic kidney disease (CKD) [1]. Hyperferritinaemia is common among patients with CKD from the Northern Territory (NT) of Australia as demonstrated by 2 recent studies showing 99% of patients on dialysis with ferritin above 500 µg/L and 92.4% with ferritin above 800 µg/L [2, 3]. Thus the use of ferritin levels to inform therapy with iron according to current national and international guidelines is challenging in this population [4].

There is no gold standard assay for measuring plasma or serum ferritin. Immunoassay methods are typically available to measure serum or plasma ferritin. These immunoassays could be broadly categorized into radiometric, nonradiometric, and agglutination assays. There is significant antibody variability in assays using antibody based assays adding to the challenges of using different methods [5]. Significant variability between results obtained using different assays would create challenges for clinical decision-making regarding iron therapy [6]. The differences in results between assays used for ferritin level measurements are recognised by the World Health Organization (WHO) which recommends more studies in this area to ensure the safe clinical use of serum or plasma ferritin levels to administer iron in patients who need it. Most of the assays are developed with calibration to WHO or other standardized international reference materials [7–9].

In the NT, different laboratories use different assays for measuring ferritin. In 2018, Territory Pathology, the main public health laboratory in the NT, changed the assay for measuring levels of ferritin and other analytes from the Abbott ARCHITECT i1000 (AA) assay (Abbott Laboratories, Abbott Park, Illinois, U.S.A, and ABBOTT, 65,205 Wiesbaden, Germany) to the Ortho-Clinical Diagnostics Vitros 7600 (OCD) assay (Ortho-Clinical Diagnostics, Felindre Meadows, Bridgend, CF35 5PZ, United Kingdom). This was at the end of the contract between Territory Pathology and the former. Immediately after implementing the change, it was noted during verification studies in the laboratories’ external Quality Assurance (QA) program and by clinical teams that the results between the two assays were up to 40% different. This change occurred at the same time as the planning of the **IN**travenous iron polymaltose for First Nations Australian (Aboriginal and/or Torres Strait Islander) patients with high **FERR**itin levels on haemodialysis (INFERR) clinical trial [10]. The INFERR clinical trial is assessing the safety and effectiveness of administering intravenous (IV) iron to First Nations Australian patients on haemodialysis with anaemia, high ferritin, and other evidence of iron deficiency [10]. The design of the trial was based on ferritin levels obtained from the AA assay. The INFERR clinical trial investigators were aware at the commencement of the trial that the change in the assay might raise questions around the cut-offs for serum ferritin levels. The protocol for the INFERR clinical trial is published elsewhere [10].

The current guidelines for the administration of IV iron to patients with kidney disease across the NT and in the INFERR Clinical trial were developed using the serum ferritin levels obtained from the AA assay (Supplementary documents 1 and 2). The ferritin cut-offs in the guidelines were determined by the results of the PIVOTAL clinical trial which provides the best evidence on the safety and efficacy of IV iron administration in haemodialysis patients [11]. Therefore, a difference between the assays would have clinical practice implications hence the need to perform an analysis to determine the appropriate cut-offs for ferritin levels using the current OCD assay. Additionally, there was a case for and need to change the cut-offs for ferritin levels in the INFERR clinical trial protocol. The INFERR clinical trial protocol inclusion criteria has serum ferritin levels of ≥ 700 μg/L & ≤ 2000 μg/L determined by the AA assay [10].

We therefore designed the trial protocol to include a sub study to assess the agreement between the two assays.

## Methods

### Study design and setting

This was a cross-sectional study comparing serum and plasma ferritin levels obtained by OCD assay to those obtained by the AA assay. Samples were obtained from the INFERR clinical trial participants (Patients on maintenance haemodialysis) and from other patients across the Northern Territory (the Top End (TE) of Australia, and Central Australia (CA)) whose testing were completed the same day on the OCD analyzers and run within 24 h on the AA analyzer.

### Serum and plasma specimens and ferritin measurement assays

Samples from the INFERR clinical trial participants were analysed. Other samples from patients whose testing were completed the same day on the OCD analyzers and run within 24 h on the AA analyzers were added to ensure wide range of ferritin levels, adding statistical strength to the comparison. The analysis of samples from the TE of Australia and CA were performed separately: 1) due to samples from the TE being available first as recruitment of participants to the INFERR clinical trial started in the TE about 7 months before starting in CA, 2) there was anecdotal evidence that ferritin levels from patients from CA were generally higher than those from the TE, and 3) there was also an imperative to get information on the comparison as soon as possible. This would inform critical clinical decisions requiring the use of ferritin level results given the potential impact the observed difference between the two assays would have on clinical decisions pertaining to the administration of iron therapy. The analyses were then performed on the combined data from the TE and CA.

The measurement of serum and plasma ferritin level was performed at the same time or within 24 h of each other on the AA and the OCD analyzers. Both serum and plasma samples were analysed because of the recognised differences in serum and plasma ferritin levels on some ferritin assays [12]. Aliquots (at least 500 µL each) were prepared from the 111 participants from the TE and 68 participants from CA. It was critical to make sure that the comparison specimens were free from significant haemolysis, icterus, or lipaemia as these could potentially interfere with the ferritin assays. The same samples were analyzed in parallel on the two analyzers.

The ferritin results were entered into Microsoft Excel spreadsheets for data management. The data were then exported to Stata software for further analysis. This study was performed in part as part of the quality improvement efforts by Territory Pathology. Territory Pathology are accredited by the National Association of Testing Authorities (NATA) in Australia [13] and participate in The Royal College of Pathologists of Australasia Quality Assurance Programs (RCPAQAP) [14].

### Statistical analysis

We used frequencies and percentages to describe categorical variables, mean (standard deviation [SD]) for normally distributed continuous variables, and median (interquartile range [IQR]) for non-normally distributed data. We used the means (SDs), kurtosis and skewness, medians (IQRs) and the Shapiro–Wilk (S-W) test for normal data to determine whether the data was normally distributed or not. (Tables 1 and 2).

**Table 1:** Summary of the distribution of the ferritin levels from the Abbott ARCHITECT and Ortho-Clinical Diagnostics Vitros 7600 assay platforms, comparison for the methods by the Passing-Bablok and Deming regression procedures and assessment of the agreement between the two methods by the Bland–Altman plot analysis

	**NT Region**		
	**TE**	**CA**	**Combined TE and CA**
Number of samples, N	*N* = 111	*N* = 68	*N* = 179
ABBOTT ARCHITECT i1000			
Mean ferritin level (µg/L)	1051.6	1190.3	1104.2
Standard Deviation	734.5	641.4	702
Skewness	0.41	-0.21	0.2
Kurtosis	3.02	2.38	2.78
Median Ferritin level (µg/L)	1171	1180.5	1171
IQR	343.6–1497.8	827.5–1628.5	510–1535
ORTHO-CLINICAL DIAGNOSTICS VITROS			
Mean ferritin level (µg/L)	650.9	726.7	679.7
Standard Deviation	469.8	385	439.9
Skewness	0.54	-0.18	0.31
Kurtosis	3.26	2.63	3.1
Median Ferritin level (µg/L)	705	763.5	731
IQR	227–906	560.5–921.5	285–908
METHODS COMPARISON			
Passing-Bablok regression^a^			
Intercept (95% CI)	-3.9 (9.56 to -0.18)	13.07 (-4.69 to 36.9)	-1.17 (-6.78 to 4.6)
Slope (95% CI)	0.63 (0.61 to 0.64)	0.59 (0.56 to 0.63)	0.62 (0.60 to 0.63)
Percentage difference ((95% CI)	37 (36 to 39)	41 (37 to 44)	38 (37 to 40)
Deming regression^a^			
Intercept ((95% CI)	-17.59 (-34.64 to -0.53)	20.74 (-2.12 to 43.6)	-6.86 (-20.91 to 7.19)
Slope (95% CI)	0.64 (0.61 to 0.66)	0.59 (0.56 to 0.62)	0.62 (0.60 to 0.64)
Percentage difference (95% CI)	36 (34 to 39)	41 (38 to 44)	38 (36 to 40)
Bland Altman			
Lower limit of agreement (%) (95% CI)	-73.75 (-77.76 to -69.54)	-66.93 (-71 to -62.85)	-71.23(-74.19 to -68.27)
Upper limit of agreement (%) (95% CI)	-24.21 (-28.32 to -20.1)	-28.79 (-32.87 to -24.71)	-25.82 (-28.78 to -22.86)
Percentage bias (95% CI)	-48.9 (-51.3 to -46.6)	-47.9 (-50.21 to -45.5)	-48.5 (-50.2 to -46.8)
Percentage Standard deviation of the bias	12.6	9.7	11.6

*CA *Central Australia*, CI *Confidence Intervals*, IQR *Interquartile range*, NT Northern Territory of Australia, TE Top End of Australia, µg/L micrograms per litre*

^*a*^*The Ortho-Clinical Diagnostics Vitros 7600 is used as the dependent variable with the Abbott ARCHITECT as the independent variable in the regression models*

**Table 2:** Shapiro–Wilk W test for normal data

Region	Variable	N	W	V	z	*p*-value
TE	Abbott	111	0.943	5.10	3.64	< 0.001
	Ortho	111	0.936	5.72	3.89	< 0.001
CA	Abbott	68	0.966	2.02	1.53	0.062
	Ortho	68	0.954	2.77	2.21	0.013
Combined TE and CA	Abbott	179	0.959	5.60	3.94	< 0.001
	Ortho	179	0.949	6.89	4.414	< 0.001

*TE *Top End*, CA *Central Australia*, Abbott Abbott ARCHITECT i1000 platform, Ortho *Ortho Clinical Diagnostics Vitros 7600 platform*, N *Number of observations*,  W: measure of how well the ordered and standardized sample quantiles fit the standard normal quantiles. 1 is perfect match for normality. V: The median values of V should be 1 for samples from normally distributed data populations. Large values of V indicate nonnormality. P-value* < *0.05 means that the hypothesis that the data is normally distributed should be rejected*

The Pearson correlation coefficient was used to measure the strength of the linear relationship between results from the two assays. However, correlation does not necessarily denote agreement.

Bland–Altman analysis (bias, mean difference and limits of agreement) was performed to compare the measurements of ferritin levels by the two assays [15]. The Bland–Altman plot was important in determining four types of data behaviour on the comparison: (1) any systematic errors (mean offset), (2) any proportional errors (the trend), (3) any inconsistent variability, and (4) any excessive or erratic variability.

The Deming regression technique was also used to compare the two assays as this technique can accommodate differences in measurement errors between the two test methods [16].

The Passing-Bablok regression for method comparison provides a non-parametric method for fitting the results from the two assays with the assumption that both assays measure ferritin levels with error. The procedure also assumes the two variables are highly correlated and have a linear relationship [17, 18].

An initial assessment of the distributions of the data from each region and then combined and by each assay was performed to determine the best regression technique. From this initial assessment, we decided to use both regression techniques as the data was not completely normally distributed. (Tables 1 and 2).

The sample types included lithium heparin plasma and serum assessed separately and compared for any differences.

All statistical analyses were conducted using Microsoft Excel (© Microsoft 2023) and Stata/MP 17.0 (StataCorp 1985–2021 StataCorp LLC, 4905 Lakeway Drive, College Station, Texas 77,845 USA).

## Results

Table 1 provides results of the distribution of serum ferritin levels, assessment of bias and agreement between the two assays. Results of the regression analysis between the two assays by the regions (TE and CA) and the combined results from the TE and CA are also presented. Table 2 summarizes the results of the Shapiro–Wilk test for normally distributed data.

### Results from the TE

As shown in Table 1, the range of serum ferritin levels from the AA assay was 3.1 µg/L to 3354 µg/L and 3ug/L to 2170ug/L from the OCD assay. The distribution of the ferritin levels by the AA assay were approximately symmetrical. The ferritin levels by OCD assay had moderately right skewed distribution. (Table 1). The S-W test showed that the data did not fulfill the criteria for normal distribution (Table 2).

**Fig. 1:**
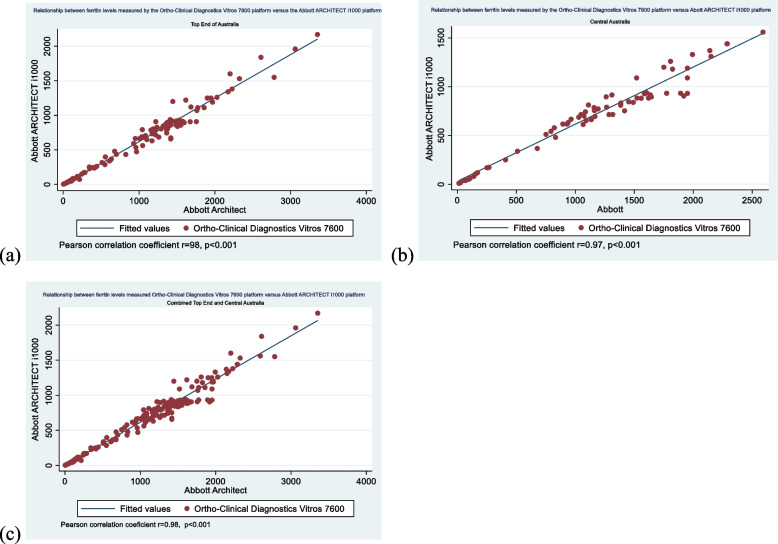
Scatter plots showing the relationship between ferritin levels as measured by the Ortho-Clinical Diagnostics Vitros 7600 platform versus the Abbott ARCHITECT i1000 platform; **a** Top End of Australia, **b** Central Australia, **c** combined Top End and Centralia Australia. Correlation is less tight at higher ferritin levels above 500 µg/L

The Pearson correlation coefficient between the two assays was r = 0.98 (95% CI (0.96–0.99, *p* < 0.001). However, there was more dispersion of the data at higher ferritin levels. (Fig. 1a).

**Fig. 2:**
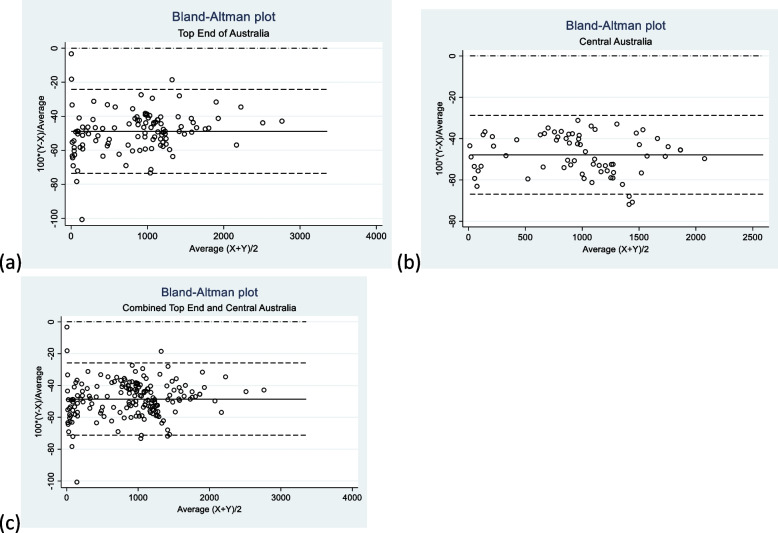
Bland–Altman plots of the ferritin levels as measured by the Ortho-Clinical Diagnostics Vitros 7600 platform versus the Abbott ARCHITECT i1000 platform, **a** Top End of Australia, **b** Central Australia (**c**) Combined data from Top End and Central Australia

The Bland Altman analysis showed a difference of -48.9% (12.6%) (95% CI; -51.3% to -46.6%). The analysis also demonstrated a proportional error across all ferritin levels by between methods comparison. The lower limits and upper limits of agreements were -73.7 (95% CI; -77.8 to 69.5) and -24.2 (95% CI; -28.3 to -20.1) respectively indicating significant variability between the methods. (Fig. 2a).

The slope from the Deming regression was 0.64 (95% CI: 0.61 to 0.66, *P* < 0.001). So, the AA assay gave results which were 36% (95% CI: 34% to 39%) higher than results from the OCD assay. (Table 1).

From the Passing Bablok regression the slope was 0.63 (95% CI: 0.61 to 0.64). Therefore, the AA assay gave results which were 37% (95% CI: 36% to 39%) higher than the results from the OCD assay. (Table 1).

When comparing plasma and serum levels, the AA assay gave the same results. However, the OCD assay results were 5% higher in serum than plasma.

### Results from CA

The range of ferritin levels from the AA assay was 14 µg/L to 2592 µg/L and 9ug/L to 1560 ug/L from the OCD assay (Table 1). The distribution of the ferritin levels by the AA assays was approximately symmetrical as were the levels from the OCD assay. (Table 1). The S-W test concurred with near normal distribution of the results from the AA assay but the results from the OCD assay were not normally distributed. (Table 2).

The Pearson correlation was r = 0.97, (95% CI: 0.96 to 0.99, *p* < 0.001) with more dispersion of the data at higher ferritin levels. (Fig. 1b).

On Bland Altman analysis, the difference was -47.9% (9.73) (95% CI: -50.2% to -45.5%), Lower and upper limits of agreement were -66.93 (95% CI: -71.01 to -62.85) and -28.79 (95% CI: -32.87 to -24.71) respectively. The results also showed similar errors as the analysis for the data from the Top End. (Fig. 2b).

The slope from the Deming regression was 0.59 (95% CI: 0.56 to 0.62). Therefore, the AA assay gave results which were 41% (95% CI: 38% to 44%) higher than results from the OCD assay. (Table 1).

The slope from the passing Bablok regression was 0.59 (95% CI: 0.56 to 0.63). This indicated that the results from the AA assay were 41% (95% CI: 37% to 44%) higher than those from the OCD assay. (Table 1).

### Results from the combined data from the TE and CA

From the combined data, the range of ferritin levels from the AA assay was 3.1 µg/L to 3354 µg/L and 3ug/L to 2170ug/L from the OCD assay. (Table 1). The distribution of the ferritin levels from the AA assay from the combined data indicated approximately symmetrical distribution whereas the levels from the OCD assay confirmed mildly right skewed distribution. (Table 1). However, the S-W test rejected the hypothesis that the results from both assays were normally distributed. (Table 2).

The Pearson correlation showed a tight correlation between the two methods r = 0.98 (95% CI: 0.98 to 0.99, *p* < 0.001) but there was more dispersion of the data at higher ferritin levels. (Fig. 1c).

The difference from the Bland Altman analysis was -48.52 (95% CI: -50.23 to -46.82). The lower and upper limits of agreement were -71.23(95% CI: -74.19 to -68.27) and -25.82 (95% CI: -28.78 to -22.86) respectively suggesting significant variability between the methods. (Fig. 2c).

The slope from the Deming regression was 0.62 (95% CI: 0.60 to 0.64). This indicates that the AA assay gave ferritin results which were 38% (95% CI 36% to 40%) above the OCD assay. (Table 1).

The Passing Bablok regression for methods comparison showed a slope of 0.62 (95% CI: 0.60 to 0.63) indicating that the results from the AA assay were 38% (95% CI 37% to 40%) above those from the OCD assay. (Table 1).

The results from the combined data were very close to either data set analyzed separately confirming the consistent differences in ferritin levels between the two assays. The observed assumption of higher ferritin for patients from CA was not confirmed by this study. Comparison between plasma and serum ferritin levels consistently indicated no difference between serum and plasma results from AA assay. However, the OCD assay results were consistently 5% higher in serum than in plasma.

## Discussion

This study demonstrates the significant challenges in using different assays to measure ferritin levels and hence using the results from these assays for clinical decision making in patients with CKD. This is more so in the NT where there are high prevalence rates of hyperferritinaemia and iron deficiency among patients with CKD and those on maintenance haemodialysis [2–4].

We used two regression techniques to compare the two assays to provide robustness to the comparison. The choice of which regression technique to use is determined by whether the data is normally distributed or skewed. The distribution of the ferritin levels from the data sets by each assay was not completely normally distributed as determined by the mean (SD), skewness and kurtosis. Additionally, the S-W test for normality generally confirmed these findings. Although the S–W test is a more appropriate method for small sample sizes (< 50 samples) and could be too sensitive to the smallest departure from normality, it can also be used to test the normality for larger sample sizes [19]. The difference between the two regression techniques is that the Deming regression assumes normal distribution of the data whereas the assumption for the Passing-Bablok regression is non-parametric. The Passing-Bablok procedure is considered by most to provide the best and most robust comparison. Both regression techniques assume that the serum and plasma ferritin levels are measured with error by both assays [16–18, 20].

Although there is very high correlation (r > 0.97) between the two ferritin measuring assays, there is significant variability between results especially at higher ferritin levels. A systematic review by Garci-Casal et.al assessed the performance and comparability of laboratory methods for measuring ferritin concentrations in human serum or plasma. They found a pooled regression coefficient of 0.985 among all methods analyzed, and 0.984 when comparing non-radiometric and radiometric methods, without statistical differences in ferritin concentration ranging from 2.3 to 1454 μg/L. They concluded that the laboratory methods most used to determine ferritin concentrations have comparable accuracy and performance. However, they did not assess the agreement between the methods. Our study is one among several which have shown clinically significant variability among methods [7, 21–25]. A study by Choy et.al. assessed the analytical bias in ferritin assays and impact on functional reference limits among five widely used commercial ferritin assays in Australia [26]. They concluded that there remained significant biases among some of the commonly used commercial ferritin assays in Australia and that more studies were needed to assess if functional reference limits are a way to overcome method commutability issues [26]. They did not have patients with CKD in their sample, but their results aligned with our findings. Ford et.al. assessed the variability of ferritin measurements in CKD and the implications of this for iron management. They found inter-method variations of up to 150 µ/L comparing six commonly used ferritin assays that evaluated thirteen pools of serum from hemodialysis and non-hemodialysis patients. They concluded that single serum ferritin values should not be used to guide clinical decisions regarding treatment of chronic hemodialysis patients with intravenous iron due to significant analytical and intraindividual variability. This is consistent with the findings from our current study [6].

There is no concordance [27] between the AA and the OCD assays meaning that ferritin results from the two assays cannot be used interchangeably in making clinical decisions. This is significant for clinical decision making in the NT where patients with CKD or on dialysis may have tests done by different laboratories using different assays depending on their location. For example, in urban Darwin, in addition to the Territory Pathology, there are other private laboratories which use different assays. This provides a challenge in decision making when samples are analyzed in one laboratory and the follow-up samples are analyzed in a different laboratory. In some remote areas in the NT, most analyses are performed exclusively by private laboratories. This makes the use of ferritin levels in interpreting iron studies when patients move between places challenging. This is more so because of the recognised need for the mobility of patients across the NT and further afield due to family connections. This was the reason why years ago, the RCPA pushed hard for doctors to be able to stipulate where certain testing could be performed. However, this was to no avail.

The AA assay gives results which are 36% to 44% higher than the OCD assay. Even with this knowledge, adjusting the results from one assay to another still provides a challenge because of the variability. Some clinicians have suggested adjusting results from one assay to another by either subtracting or adding 36% to 44% to the results. However, this adjustment to harmonize the results, though plausible, will need robust statistical testing for accuracy and clinical relevance [28].

The bias between the two assays is also as high as 49%. This is consistent with findings from other similar studies comparing other ferritin assays although the degree of bias may be different [6, 26]. This high level of bias provides a significant challenge for people interpreting results using the different assays. This suggests that the interpretation of results from the different assays should ideally not be used for making clinical decisions.

As described above, several methods are available for measuring ferritin, but studies have shown poor comparability among the assays [6, 7, 9, 26]. With the lack of a gold standard assay, in order to improve the comparability between the assays, the WHO Expert Committee on Biological Standardization established international reference materials to which all the developed assays are referenced [7–9, 28]. The WHO recommendation is that all methods are acceptable if a commutable material traceable to the WHO international reference standard is used to calibrate the assay [9, 28]. The WHO also recommends that one method does not appear to be superior to another. However, they strongly recommend that once a method has been selected, as much as possible, that same method should be used for the follow-up of individuals and populations. This will be challenging in the NT setting where the availability for several different laboratories is inevitable.

This heterogeneity in ferritin measurement methods creates challenges in clinical decisions in iron deficiency and overload. The challenges are further amplified in inflammatory states and in people with CKD both of which are highly prevalent in the NT [4] where levels of serum ferritin are generally higher than the reference ranges determined by the WHO and other guidelines. Performance of different methods need to be evaluated to determine clinical decision limits in people with CKD.

From a clinical care point of view, the results have prompted the need to change the ferritin cut-off in guidelines for managing IV Iron administration in haemodialysis patients in the NT. The findings from this study have led to adjustment of the ferritin cut-off levels within the clinical guidelines used to administer iron (Supplementary documents 1–4). This adjustment was limited to the lower cut-off of 500 µg/L for the INFERR clinical trial (this is the higher cut-off for the standard clinical care guideline) as adjusting the higher cut-off of 2000 µg/L for the trial was determined not to make any significant difference by the INFERR clinical trial Trial Management Committee (TMC).

### Limitations of the study

There were some limitations to the study. First, the clinical decision points for ferritin levels in a population with hyperferritinaemia and high prevalence of CKD were not determined. This was beyond the scope of the study. It is part of the next phase of this work to determine these points and the critical values from the analysis within the INFERR clinical trial and included in the analysis will be data from other assays used in laboratories across the Northern Territory. Second, although the data came from this multi-centre study with a reasonable sample size, this was from the one region (the NT of Australia), and predominantly from First Nations Australians on haemodialysis so the results may not be generalizable to other populations. However, the results are similar to findings from other studies with different populations [26]. Third, the comparison was limited to two assays used by the NT public laboratories whereas there are other assays used by other private laboratories. A similar study is being planned as a collaborative amongst all laboratories operating within the NT to have a clarity of their differences.

## Conclusion

In making clinical decisions regarding the management of iron deficiency in patients with CKD or on maintenance haemodialysis, it would be important to use ferritin results from the same assay. If the laboratory changes the assay used, it is essential to assess the degree of agreement between results from the old assay and the one the laboratory would have changed to. This is a routine component of all Australian laboratories’ accreditation with NATA. Assay changes must be notified and covered off as per the National Pathology Accreditation Advisory Council (NPAAC) and International Organization for Standardization (ISO) requirements. Further studies to harmonize ferritin assays are required.

## Supplementary Information

**Additional file 1.**

**Additional file 2.**

**Additional file 3.**

**Additional file 4.**

**Additional file 5.**

## Data Availability

All data supporting the study are presented in the manuscript and available on reasonable request to the investigators through correspondence author.

## Abbreviations

AA: Abbott ARCHITECT i1000
CA: Central Australia
CAHREC: Central Australia Human Research Committee
CI: Confidence intervals
CKD: Chronic kidney disease
DSMB: Data Safety and Monitoring Board
HREC: Human Research Ethics Committees
INFERR: INtravenous iron polymaltose for First Nations Australian patients with high FERRitin levels on haemodialysis
IQR: Interquartile range
ISO: International Organization for Standardization
IV: Intravenous
MSHR: Menzies School of Health Research
NATA: National Association of Testing Authorities
NHMRC: National Health and Medical Research Council
NPAAC: National Pathology Accreditation Advisory Council
NT: Northern Territory
OCD: Ortho-Clinical Diagnostics Vitros 7600
QA: Quality Assurance
PIVOTAL: Proactive IV irOn Therapy in haemodiALysis patients
SD: Standard deviation
SSA: Site specific assessment
S-W: Shapiro–Wilk W test for normal data
RCPAQAP: The Royal College of Pathologists of Australasia Quality Assurance Programs
RGO: Research Governance office
TE: Top End of Australia
TMC: Trial Management Committee
WHO: World Health Organization
