# Supplementary material for: Comparison of two ferritin assay platforms to assess their level of agreement in measuring serum and plasma ferritin levels in patients with chronic kidney disease
Source: BMC Nephrol. 2023 Jun 30;24:198. doi: 10.1186/s12882-023-03255-6 (PMC10314376; doi:10.1186/s12882-023-03255-6)
Supplement: Supplementary file 5 — Additional file 5. [file 12882_2023_3255_MOESM5_ESM.docx]

The following are members of the INFERR study group.

Sandawana William Majoni, Jane Nelson, Darren Germaine, Libby Hoppo, Stephanie Long, Shilpa Divakaran, Brandon Turner, Jessica Graham, Sajiv Cherian, Basant Pawar, Geetha Rathnayake, Bianca Heron, Teana Brewster-O’Brien, Louise Maple-Brown, Robert Batey, Peter Morris, Jane Davies, David (Kiran) Fernandes, Madhivanan Sundaram, Asanga Abeyaratne, Yun Hui Sheryl Wong, Paul D. Lawton, Sean Taylor, Federica Barzi and Alan Cass.

The following are members of the INFERR DSMB

David Wheeler, Alex Brown, Martin Gallagher and Zhiqiang Wang.
